# Local classifications of fever and treatment sought among populations at risk of zoonotic diseases in Ghana

**DOI:** 10.1371/journal.pone.0201526

**Published:** 2018-08-23

**Authors:** Fidelia Ohemeng, Jesse Sey Ayivor, Elaine Tweneboah Lawson, Yaa Ntiamoa-Baidu

**Affiliations:** 1 Department of Sociology, University of Ghana, Legon-Accra, Ghana; 2 Institute of Environment and Sanitation Studies, University of Ghana, Legon-Accra, Ghana; 3 Centre for African Wetlands, University of Ghana, Legon-Accra, Ghana; 4 Department of Animal Biology and Conservation Science, University of Ghana, Legon, Accra, Ghana; Wistar Institute, UNITED STATES

## Abstract

In the past four decades, there has been an increase in the occurrence of zoonotic diseases. Some outbreaks have been devastating because of the inability of individuals and health workers to identify the diseases early. Generally, most zoonotic diseases are heralded by a fever. While fevers are common, they are often the symptoms of different diseases. This paper explores how a population at potential risk of zoonotic diseases identify fevers, and what treatments they seek when they develop fevers. The data are from focus group discussions and a survey of three communities in the Brong Ahafo, Volta and Greater Accra regions in Ghana. The quantitative data were analysed using descriptive statistics while the qualitative data were analysed using thematic analysis. The findings indicate that the perceived causes of fever differ from the traditional biomedical view. While orthodox treatment was the preferred choice for most participants, rural dwellers utilised traditional medicine more than their urban counterparts. Though there is no record of bat-borne zoonotic disease in Ghana, our findings could be used as a proxy to indicate how populations at risk of exposure might respond in the event of a spillover event from a zoonosis. We recommend that educational campaigns on zoonotic diseases should target rural dwellers, especially farmers, who may be most at risk of zoonoses.

## Introduction

In the event of an outbreak of an infectious disease, the success in reducing morbidity and mortality depends on early detection and rapid response [1] by health officials. This means that the affected are able to identify the disease correctly and seek swiftly the appropriate treatment [2]. There are instances where delayed detection and misdiagnosis of disease outbreaks, because they usually resemble an already known disease and/or that the disease is not known to be common in that country or region, have led to serious public health implications [1]. For instance, when an encephalitic disease first broke out in Malaysia, it was erroneously thought to be Japanese encephalitis, which is a viral disease and associated with pigs in South East Asia. However, the clinical and epidemiologic characteristics of the disease suggested otherwise. Henipah virus was only detected after analysis of cerebrospinal fluid from several patients [3–5]. According to Looi and Chua (p.64 of [4]), the “conflicting governmental pronouncements that Japanese encephalitis was the culprit delayed appropriate action for outbreak control”. Similarly, a major issue that affected an Ebola Virus Disease (EVD) outbreak in West Africa was weak surveillance, which led to delayed detection and misdiagnosis. Thus EVD, which started in December 2013 in Guinea, was officially brought to the attention of the World Health Organisation (WHO) in March 2014 [5], three clear months after the disease outbreak, by which time it had spread to other districts and neighbouring countries, such as Liberia and Sierra Leone. The delay in notifying the WHO was related to misdiagnosis and a delay in identifying the disease. Similarly, health officials at a hospital in Dallas, Texas admitted that they misdiagnosed a patient who presented with EVD symptoms at the facility, which led to the death of the patient [6].

Generally, fever is the most common symptom of zoonotic diseases [3, 5, 7]. During the henipah virus outbreak in Malaysia, 110 patients were admitted to hospital between February and June 1999. The symptoms presented by patients included fever (97%), headache (65%), dizziness (36%), vomiting (27%), reduced level of consciousness (21%) and non-productive cough (14%) [3]. Out of 110 patients, 94 were confirmed to be infected with henipah virus. Similarly, symptoms commonly presented in the 2013 EVD outbreak in West Africa included fever (87.1%), fatigue (76.4%), loss of appetite (64.5%), vomiting (67.6%), diarrhoea (65.6%), headache (53.4%), and abdominal pain (44.3%) [5].

Fever is very common in developing countries. The presence of a fever may be an indication of an infection and thus often seen as the symptom of diseases such as malaria, pneumonia and typhoid fever [8–10]. In addition, fevers are normally viewed as a proxy for childhood diseases [11].

There are several cross-cultural variations in how fevers are perceived [12, 13]. In some parts of Africa, the perception and causes of fevers are often different from the biomedical views of diseases. In one study on the local classification of fever in a malaria endemic region in Uganda, Nsungwa-Sabiiti et al. [13] found that fever was classified in many ways which were different from the biomedical views of fever, and that only one ‘fever of the mosquito’ (*omutsutsa owe miibu*), was rightly described as the biomedical symptom of malaria. In a study on malaria-related beliefs in a rural area in Ghana, Ahorlu et al., [12] report that fever and malaria, which are locally known as *asram* or *atridii*, were used interchangeably to refer to the same thing. This example shows that fevers may be poorly understood. In this paper, we report on the beliefs of communities that live in close proximity to certain wildlife, such as bat roosts in Ghana, regarding the causes of fever and the treatments they seek. These populations are considered to be at potential risk of zoonotic diseases, because a number of viruses, including henipah-like virus, have been found in bats and pigs in Ghana [14, 15]. Bats are associated with zoonoses with significant global public health impacts, and believed to be reservoirs of filoviruses (e.g., EVD and Marburg viruses), paramyxoviruses (e.g., Hendra and Nipah virus) and lyssaviruses (e.g., Rabies virus, Lagos bat virus, etc.) [16, 17].

## Data collection methods

### The study sites

**Fig 1:**
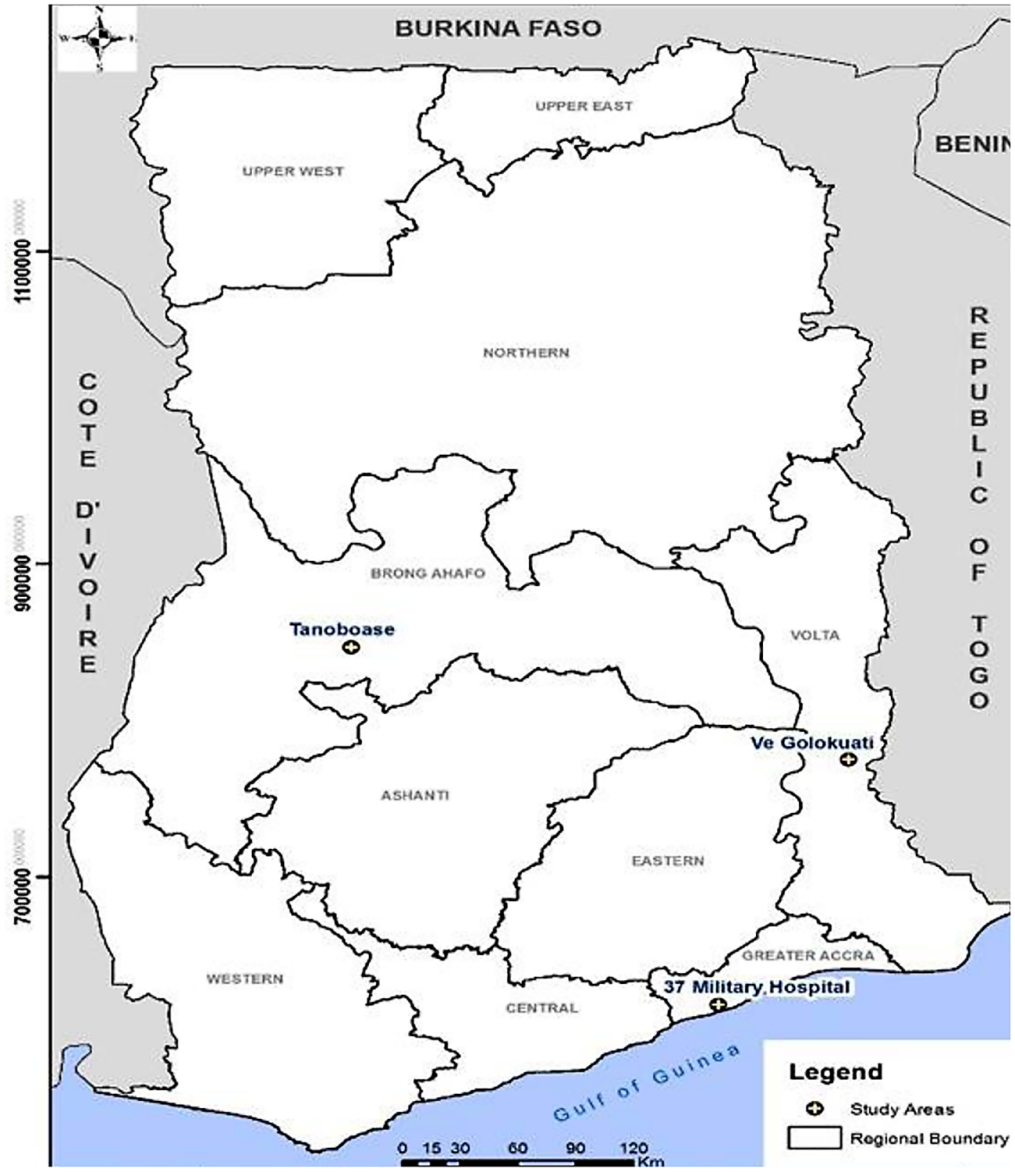
Map showing the study sites.

The study was conducted in three communities from three regions in Ghana—two were rural and one urban. The rural communities were Tanoboase in the Brong Ahafo Region and Ve Golokuati in the Volta Region and Accra, in the Greater Accra region (Fig 1). The communities were chosen because of their close proximity to large bat roosts, mainly *Eidolon helvum* and *Epomophorus gambianus*. At Tanoboase, the bats roost in a 3,000-acre sacred forest and feed predominantly on fruit trees, especially cashew, pawpaw and mango on farms in and around the village. Human-bat interaction occurred when people go to the forest to hunt and also through processing and consumption of bats. In addition, residents were exposed to bat faeces to both their hands and feet when they visit their farms. At Accra, bats roost in large mahogany and neem trees at the 37 Military Hospital, the Department of Parks and Gardens and the 37 Military Barracks in Accra, which are all close to each other. People at the hospital, residents of the barracks, workers at the Parks and Gardens and commuters at the nearby lorry station are all exposed to bat droppings and urine in their daily activities. Bat droppings were visible on the ground, the walls and roofs of the buildings. At Ve Golokuati, bats roost on large trees such as mango and neem trees in the township and nearby forests. Bat roosts could be found at the market, homes, schools, the clinic and the chief’s palace. Residents were exposed to bats droppings directly when the bats defecate onto their wares at the market, receptacles for harvesting rain water, their food and clothing.

### Research design

The tools for data collection were based on questionnaires and focus group discussions.

#### Quantitative design

At Tanoboase and Ve Golokuati, sampling involved one participant from every fifth household being selected. At Accra, the population consisted of health workers at the 37 Military hospital, workers at the Department of Parks and Gardens and residents at the 37 Military Barracks. The total sample for this area was divided into three equal parts of 30 or more respondents. Due to the fact that this population consisted of workers who mainly run shifts and were very busy, accidental sampling was used. Thus, anyone who was available and willing to take part in the study was included. All participants were interviewed face to face (S1 Appendix). The questionnaire was both open-ended and close ended. The questions included demographic characteristics, assets owned, hunting, processing and consumption of bats, conservation of bats and incidence of fevers and treatment sought. This particular paper is based on questions on the occurrence of fevers and the treatment sought. In the survey, every resident 15 years and above qualified to be part of the study. The data were cleaned, coded and entered into the Statistical Package for Social Sciences (SPSS version 21). The data were analysed using frequencies and percentages and chi-square tests to determine relationships between some variables.

#### Qualitative methods

The main qualitative instrument used was focus group discussions (FGDs). A total of six FGDs were conducted—two in each community. There were separate FGDs for men and women. Though the topic under discussion was not a sensitive one, traditional women generally do not speak in the presence of men [18]. In all, 192 participants took part in the FGDs. At Tanoboase, 98 women and 38 men took part in the study, 15 women and 21 men at Ve Golokuati, and 6 females and 14 males at Accra. The high number of people who took part in the FGDs in Tanoboase was due to how the participants were recruited. As part of the community entry, we met the traditional leaders in the rural communities, who were briefed about the study and their support was sought. On the day of the FGDs, an announcement was made on the public address system in the community where members were informed to come to the community social centre where the researchers were located. Scores of people came out to be part of the study. The team could not send them away because that would have been considered offensive. However, the high number of participants did not affect the quality of the discussions as they were facilitated by one of the researchers and two research assistants. Moreover, the researchers made sure to include interest groups such as hunters, butchers, farmers, traders, and traditional healers in the discussions. Apart from these interest groups, the criteria for inclusion in the FGDs were that the individual should be 18 years and above and be willing to take part in the study. The FGDs were tape-recorded and later transcribed and translated into English. The data were analysed based on themes as described by Braun and Clark [19]. This included familiarity with the data, and identifying and grouping into themes.

One limitation of this study was that the populations of the three communities were not known. This affected the determination of the sample size and the sampling frame used for the study. Based on the criteria that a rural area has a population of not more than 500 people, we decided to sample an estimated 20% of the population, with the sample size of 340.

To ensure external validity, several steps were taken in the design. As already indicated above, wildlife such as bats can be found all over the country, but widely consumed in the southern sector. The southern sector was categorised into three—the forest zone, the coastal and the Volta zone. The Volta region mimics all four ecological zones in Ghana. Tanobaose represented the forest zone, Ve Golokuati the Volta, while Accra represented the coastal belt and was also cosmopolitan in nature. Furthermore, the three areas had different levels of proximity to known bat roosts. In Tanobaose, residents did not interact with bats except when they visited the forest to hunt or came into contact with bat droppings on their farms. At Ve Golokuati and Accra, bats roost in the homes, offices, markets and schools. Thus, the findings of this study may be generalised to some populations that live in close proximity to bat roosts but not to the larger Ghanaian population.

Ethical clearance for the study was obtained from the Institutional Review Board (IRB) of the University of Ghana situated at Noguchi Memorial Institute for Medical Research. The IRB details are as follows: Study Number 002/13/14, Internal Number 1796. The consent of participants was sought before information was acquired. Those who agreed to take part in the study signed the consent form. Those who could not write used a thumbprint on the consent form. Though the study was part of the larger Dynamic Drivers of Diseases in Africa Consortium, we did not need the permission of DDAC to publish the data since the researchers are part of the consortium.

## Findings

The three hundred and forty (340) respondents who took part in the survey comprised of 126 from Accra, 111 from Ve Golokuati and 103 from Tanoboase. There were 164 females representing 48.2% of the respondents and 174 males making up 51.8%. While 12% had post-secondary education, 9% had tertiary education and 15.9% had no formal education. The economic activities engaged in by the respondents included farming, trading, dressmaking, hairdressing, artisanal work, construction, teaching and military/health workers. Among the rural population, the predominant occupations were farming and trading. The household sizes ranged from one to 15, with 45.7% with households of between one and five and 43% had household size from six to 10. Generally, household sizes in the two rural areas were larger compared to the urban site. While 53.5% of respondents in Tanobaose and 47.9% in Golokuati had household size of six and above, only a third of the respondents at Accra had household sizes above six. Generally, household sizes at Accra ranged from one to five members (Table 1)

**Table 1:** Demographic characteristics of respondents from survey.

	Tanoboase	Ve Golokuati	Accra	N	%
***Age***					
15–25 years	15	10	6	31	9.1
26–35 years	25	18	42	85	25.0
36–45 years	19	21	37	77	22.6
>45 years	44	62	41	147	43.2
***Sex***					
Female	52	65	47	164	48.2
Male	51	46	79	174	51.8
***Education***					
No Education	39	9	6	54	15.9
Primary	14	12	8	34	10.0
JHS^1^	25	25	19	69	20.3
SHS^2^	6	15	27	48	14.1
Post-Secondary	4	18	20	42	12.4
Tertiary	2	9	20	31	9.1
Middle School	13	23	26	62	18.4
**Household size**					
1–5	32	37	59	128	45.7
6–10	51	45	26	122	43.6
>11	13	12	5	30	10.7
**No. of assets owned**					
<3	50	46	10	106	31
4–5	44	44	72	160	47
>6	9	21	44	74	22

^1.^ Junior High School.

^2.^ Senior High School.

It was a challenge obtaining information on incomes, especially in the rural areas. In the absence of data on income and expenditure, household assets were used as an indicator of the income of individuals. The assets used to indicate the wealth of respondents were made up of nine items. These included mobile phones, bicycles, motor cycles, television sets, sewing machines, cars, internet accessibility, refrigerators, radios and land. While land and the type of building materials could also be used in measuring wealth [20], in this study they were not used in measuring the wealth of respondents because land ownership did not play the same role in livelihood activities in all the study sites. While in the rural areas land played an important role in livelihoods, urban dwellers did not require land for their sustenance. In addition, the materials used in building houses differed greatly for rural and urban areas. In the rural areas, many houses were built of mud, with a thatched roof, whiles in the cities, houses were built of cement blocks and the roofs were mostly of corrugated iron sheets. This difference made comparing land ownership and building type between rural and urban areas problematic. To measure the wealth of participants, we summed up the number of assets of each participant. The number of assets owned was categorised into three—less than three, between four and five, and six and above. Participants with more than six assets were categorised as wealthy, this was followed by participants with four or five assets, and then those who had three and less assets, in that order. Of the participants, 31.2% had < 3 assets, 47.1% had between four and five assets and 21.8% had >6 assets. The mean, mode and median number of the assets owned indicated that the majority of the participants had an average of four assets.

### Types of illness experienced

In the survey, the illnesses and complaints that participants commonly experienced were malaria (23.8%), fever (15.6%) and body pains (11.5%). Others mentioned included hypertension (4.4%), headache (2.4%), skin diseases (1.8%) and rheumatism (1.8%) (Table 2). Accra, which is an urban area, reported more malaria cases (67%) than Tanoboase (17%) and Golokuati (16%). The diseases reported in the FGDs were similar to those reported in the survey. There were two diseases that were reported in the FGDs but not in the survey. These were piles/hemorrhoids (*kooko*) and diabetes. It is worthy to note that, in Ghana, *kooko* is believed to be the root cause of many diseases. Many herbal drug peddlers claim to have an antidote (drugs) for piles, which they claim could heal most diseases as well.

**Table 2:** Types of complaints experienced.

Disease	N	%
No response	121	35.6
Malaria	81	23.8
Fever	53	15.6
Body pains	39	11.5
Hypertension	15	4.4
Headache	8	2.4
Skin disease	6	1.8
Rheumatism	6	1.8
Diabetes	3	.9
Asthma	3	.9
Sickle cell	2	.6
Typhoid Fever	2	.6
Anaemia	1	.3
**Total**	**340**	**100.0**

### Beliefs about the causes of fevers

The information on the beliefs of the causes of fever were solicited from the FGDs. At Tanoboase and Accra, fever is known locally as *atridii*, *abunu* or *feber*. *Feber* is the corrupted form of fever. At Ve Golokuati, fever is locally known as *asra*. The symptoms of fever as mentioned by participants included general bodily weakness, colds, high body temperatures, shivering, vomiting, yellowish urine, weak knees and headaches. The participants further distinguished between two types of fever: “normal fever”, whose symptoms are as indicated above and “high fever”, locally known in Twi as *krakran*. High fever, they submitted, is characterised by "irrational behaviours as if the sufferer has a mental health problem”. They further indicated that high fever is caused by changes in the blood of a person. One participant in the FGDs intimated that: "when the blood increases, it causes high fever; the high fever then affects the brain, which makes the patient behave as if he/she has a mental illness".

Further, it was believed that fever occurred when one consumed too much oil or is exposed to the sun for long periods and too much consumption of mangoes. According to respondents, butterflies bore into the mangoes and lay eggs in them, which results in the development of tiny worms in the mangoes. They claimed that children especially could develop fever if they ate the worm-infested mangoes. Another cause of fever is the eating of new yams. As a participant submitted: “when new yam comes, fever is common”. It was believed that fever is normally common during certain times of the year and not others. Participants believed that fever is common between the months of June and July and during the harmattan season which occurs between December and February. June/July is the peak of the rainy season in Ghana and it is during this period that a lot of insects, such as mosquitoes, breed. It is during this period that fever was assumed as a symptom of malaria.

### Frequency of fever

Of the 340 respondents in the survey, 75.6% (238) indicated that they had experienced fever, and 24.4% (77) indicated they did not. Participants at Ve Golokuati reported more fever (42.2%) than Accra (31.9%) and Tanoboase (25.6%). Of those who reported that they have experienced fever, 57.9% indicated they experience fever occasionally, 30.8% indicated not very often and 4.5% indicated once every week (Table 3). We wanted to find out whether the economic activity people engaged in exposed them to fever. The findings show farmers reported the most fever episodes (42.6%) compared to other professions (11.4%). A chi-square test was performed to examine the relationship between economic activity and the occurrence of fever. Considering the majority of the respondents were farmers, we re-coded the economic activities into farming and non-farming. A statistically significant relationship was found between economic activity (farming and non-farming activities) and occurrence of fever (X^2^ = 4.20, N = 284, df = 1, p = 0.039).

**Table 3:** Frequency of fever by community, economic activity and rate of recurrence.

Study site	Experience of Fever (Yes)
Ve Golokuati	42.4% (101)
Accra	31.9% (76)
Tanoboase	25.6% (61)
**Total**	**238**
**Economic activity**	
Farming	86 (42.6%)
Trading	26.7% (54)
Artisanal work	19.3% (39)
Formal sector workers	11.4% (23)
Total	**202**
**How often participants experience fevers**	
Once a week	4.5% (10)
Twice a week	1.8% (4)
Every month	2.7% (6)
Every 2 months	2.3 (5)
Occasionally	57.9% (128)
Not often	30.8% (68)
**Total**	**221**

The data were further analysed to examine the relationship between income levels (which by proxy are the assets owned) and the experience of fevers as suggested in the literature [11]. Respondents with more than six assets reported the least fever episodes (20.6%). The difference in the various income levels was, however, not statistically significant (X^2^ = 2.131, N = 315, df = 2, p = 0.345).

### Health seeking behaviours

**Fig 2:**
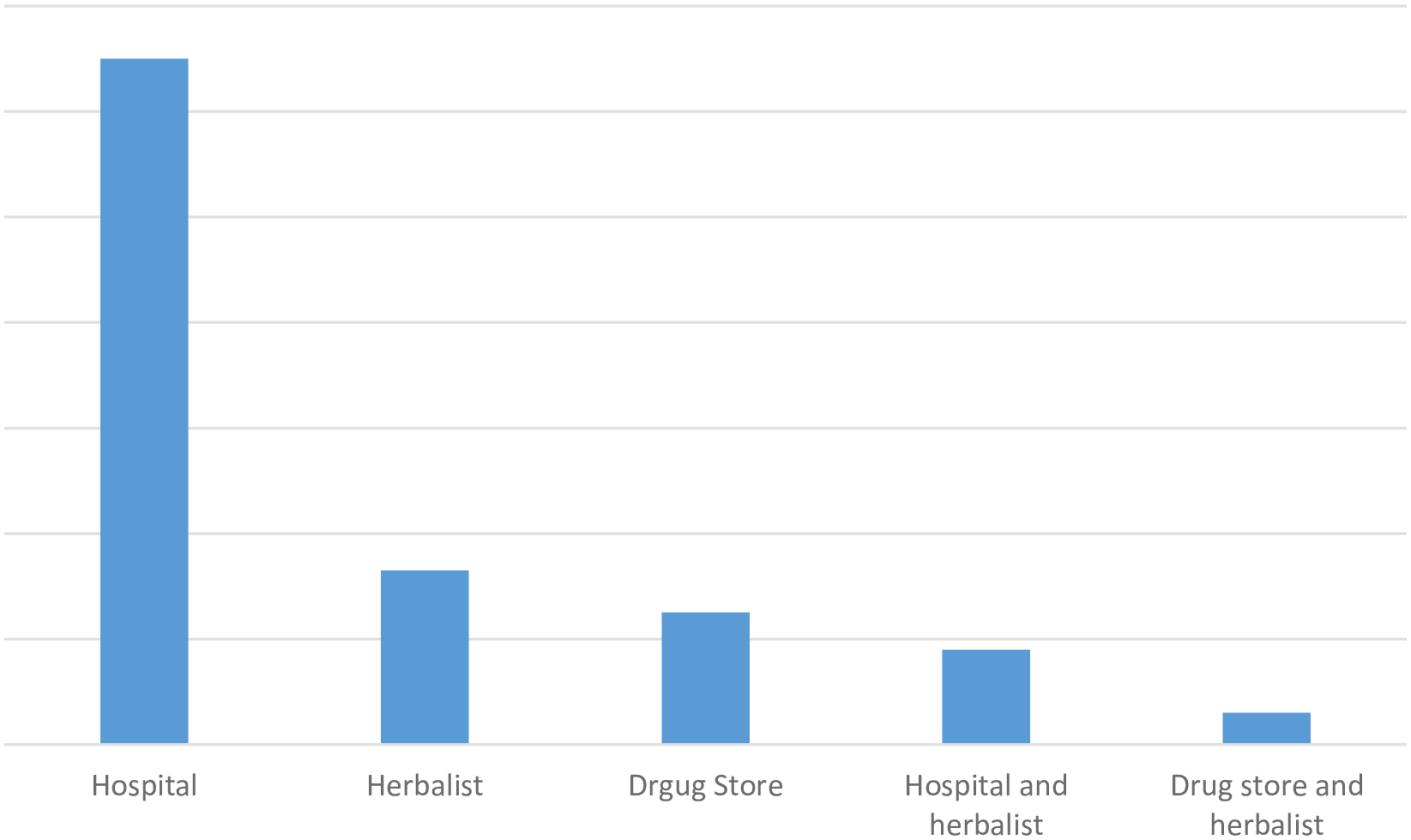
Types of health seeking behaviour.

The study also investigated the type of treatment respondents sought when they experienced fever. Of the 212 responses 59.9% sought treatment at a hospital, 15.2% used herbs, 11.5% visited the drugstore, 8.3% and 2.8% used a combination of hospital and herbs and drug store and hospital, respectively (Fig 2).

#### Community and health seeking behaviours

The type of treatment sought for fever varied by community. While 39.2% of respondents at Golokuati sought treatment at a health facility, 31.5% and 29.2% sought treatment at a health facility at Accra and Tanoboase, respectively. Generally, the rural dwellers tended to use herbs more than urban dwellers. In all, 33 participants indicated that they used herbs. Of that number, 16 (48.5%) were recorded from Ve Golokuati, 12 (36.4%) from Tanoboase and five (15.2%) from Accra (Table 4). Indeed, in the FGDs, participants at Tanoboase and Golokuati exhibited more knowledge in medicinal plants than those at Accra. Some of the plants reported to have medicinal value included *moringa*, neem tree, guava, cinnamon, India tree, dry cocoa leaves, pawpaw leaves, pear, and mango. They indicated further that the leaves, bark, roots or other parts of trees can be boiled and the resultant decoction drunk. As an example, this is what one FGD participant said:

“I do not remember the last time I was sick. I constantly drink herbal medicine brewed from militia and neem plants. The male workers at the other side are constantly brewing herbal concoctions so we go and drink some.”

**Table 4:** Health seeking behaviours by the community.

	Hospital	Herbalist	Drug Store	Hospital and Herbalist	Drug Store and Hospital	Total
Community						
Tanoboase	38	12	9	3	1	63
Ve Golokuati	51	16	10	12	5	94
Accra	41	5	6	3	0	55
Total	130	33	25	18	6	212

According to respondents, the herbal concoction induces one to pass urine frequently and that through frequent urination, the disease leaves the body. Indeed, in the survey, when respondents were asked whether they knew of any traditional method of healing, the two rural communities had the highest number of respondents who indicated that they knew some traditional healing method. Ve Golokuati had the highest number of respondents who had knowledge of herbs (65.3%), followed by Tanoboase (33.3%) and Accra (1.3%). The total number of respondents who had some knowledge of herbal medicine was 75. Of the 75 respondents, 36 indicated that herbal medicine could be used to cure fever, while 23 said it could be used to cure malaria.

#### Livelihood and health seeking behaviours

The data indicate that more than 40% of the respondents who used hospital facilities were farmers, whereas 25% were traders. This was followed by artisans (20%) and the military/health personnel and teachers. In addition, farmers tended to use herbs (41%) more than traders and artisans. However, a chi-square test showed that the relationship between the economic activity one engaged in and the type of treatment sought for fever was not statistically significant (X^2^ = .561, N = 187, df = 3, p-value = 0.905). Moreover, there was no statistically significant association between the number of assets owned and the kind of treatment used (X2 = 7.737, N = 212, df = 6, p-value = .258).

## Discussion

In this paper, we used beliefs about the causes of fever and the treatment sought as a proxy to explore how a population at risk of potential zoonotic diseases might react in the event of a zoonotic disease outbreak. The fact that malaria was the most common disease reported is not surprising, as it is endemic in Ghana [10, 21]. Malaria accounted for more than a third of all outpatient department (OPD) cases and almost half of hospital admissions in children under five years [21]. In adults, malaria leads to the loss of productivity and welfare [22]. The observation that malaria occurs more among urban dwellers than rural dwellers is inconclusive. While some studies contend that malaria is more endemic in urban areas [23, 24], others argue that malaria is more prevalent among rural dwellers [25–27]. Data from the 2014 Ghana Demographic and Health Survey [28] indicate that the prevalence of childhood malaria was higher in rural areas (52.9%) than in urban areas (16.9%).

We found that the perceptions about the causes of fever differed from a biomedical view. In biomedicine, a fever may be caused by an infection and is often a symptom of diseases such as malaria, pneumonia and typhoid [29]. In this study, only a few of the participants indicated that fever may be caused by infected vectors, such as mosquitoes and could be the symptom of malaria and other diseases. In a study on the local classification of fever in a malaria endemic region in Uganda, Nsungwa-Sabiiti et al., [13] report of the varied classification of fever, many of which were different from the biomedical view. Of the different classifications reported in their study, only one rightly described the biomedical symptom of malaria.

In this study, it was believed that fever is caused by eating certain foods such as mango and new yam and from long exposure to sunlight. Similar findings were reported in the studies of Ahorlu et al., [12]. In a study of the local beliefs and practices associated with malaria in Ghana, Ahorlu et al. [12] indicate that the term *atridii* and *asra* were used interchangeably to refer to fever and malaria. In our study, the terms used to refer to fever—*atridii*, *feber* and *abunu*—were not the same for malaria. In their study, the perceived causes of fever as indicated by their participants were similar to the perceived causes of fever in the present study. In both studies, fever was believed to be caused by eating un-ripe mangoes, excessive exposure to sunlight and the consumption of fresh yams. Clearly, there are no data to support the assertion that fever can be caused by eating un-ripe mangoes and fresh yams.

Furthermore, we investigated participants’ views on what time of year they think fever is common. This information is important to establish whether there may be an association between the time that certain animals such as bats are known to return from migration and the time that fever is thought to be common. While many local bat populations are believed to increase between September and December and decline between March and April, fever is reported to be most common between June and July and during the harmattan season. This means that fever is common when the bats have not returned from migration. Moreover, the participants did not associate bats with any diseases [30]. They contend that no disease outbreak has been associated with bats. Indeed, there is no record of a bat-borne disease outbreak in Ghana, in spite of the fact that human-bat interactions are very common [30, 31]. It is possible that Ghana has not recorded a bat-borne disease outbreak because none has occurred, surveillance is inadequate, or that the local populations may have developed immunity in response to repeated exposure to bats. As reported in Gilbert et al. [32] study of populations in the Amazon Basin exposed to rabid vampire bats, the repeated exposures of the local populations may have led some to develop appropriate immune responses.

The fact that farmers tended to experience fevers more than people in other occupations may be insightful. Livestock farmers tend to be exposed to diseases due to their contact with domestic and wild animals, which harbour a variety of pathogens [33]. For instance, a study in a population in the Amazon Basin exposed to vampire bats showed that farmers who owned animals bitten by bats were at risk of developing rabies [32]. Likewise, crop and fruit farmers tended to be exposed to insect bites and animal secretions, more than people of other occupations, and may be exposed to different disease pathogens. Farmers therefore, may be more at risk of zoonotic diseases than people of other occupations.

In this study the participants identified a second type of fever, which they termed high fever. This, they claimed is caused by a sudden rush of blood to the brain, making the sufferer to behave abnormally. This type of fever is reported to be common in children. Some children experience febrile seizures and convulsions when they develop high body temperatures due to the presence of a virus or bacteria [34]. However, febrile seizures in adults are not common. The type of high fever described by the participants are characterised by talking to oneself, shouting and other strange behaviours.

The study also explored the types of treatments sought by participants when they experience a fever. These are orthodox, herbal and over the counter drugs, and a combination of all three. It is refreshing that majority of the participants reported that they sought treatment at a health facility. The fact that more people at Ve Golokuati, which is a rural community, sought treatment at a hospital than Accra is surprising. The site at Accra is a major referral hospital, and is close to other major hospitals such as the Police and Ridge hospitals. However, rural dwellers tended to use herbal medicine more than urban dwellers. This might be due to structural challenges such as unavailability of a health centre as is the case at Tanoboase and the adherence to local customs and traditions. There was no health facility at Tanoboase—the closest health facility was at Tuobodom, about 5.9 km from Tanoboase. In the FGDs, the majority of the residents indicated that they normally visited the Holy Family Hospital at Techiman, which is about 12.5 km away. They pointed out that they preferred the Holy Family Hospital to the clinic at Tuobodom because it is the largest hospital in the district and therefore if there was an emergency that is where they would be referred. Though Golokuati had a health centre, it had the highest number of respondents who said they used herbal medicine.

## Conclusion

The upsurge of zoonotic diseases within the past four decades poses a serious threat to human populations. Successfully identifying causes and symptoms is key to the prevention and treatment of such outbreaks. The challenge, however, is when a new and emerging disease has similar characteristics to an already known disease. How that disease is categorised, perceived and the treatment sought is critical to how the new disease would be managed. In this study, we explored how fever, which is a common symptom of many tropical and zoonotic diseases, is perceived and managed by three Ghanaian communities at risk of potential zoonotic diseases.

The study has revealed that the perceived causes of fever are different from the biomedical view. Given the role of wildlife in the transmission of zoonotic diseases and the fact that there are regular human-wild animal interactions in Ghana, there should be public health education to encourage people who frequently interact with wildlife to seek immediate medical attention during a fever episode. Educational programmes on the emergence of zoonotic diseases should target farmers, since they could be the potential source of a disease outbreak. In addition, public health education should be crafted in part based on the beliefs of the people.

The study would have been enhanced if the results of blood samples taken from participants, livestock and pets were available. Indeed, blood samples were collected from the participants and domesticated animals and pets. However, there were challenges with the analysis due to the unavailability of laboratory equipment in Ghana. As already indicated above, henipahvirus had been detected in the blood sample of pigs at Ve Golokuati (15), so there is the need for further tests to determine whether this is widespread or an isolated case.

## Supporting information

S1 Appendix Questionnaire of the study. (DOCX)

S1 File STROBE checklist. (DOC)

## References

[1] Woolhouse ME, Rambaut A, Kellam P. Lessons from Ebola virus disease: Improving infectious disease surveillance to inform outbreak management. Sci Transl Med. 2015;7(307):307rv5. 10.1126/scitranslmed.aab0191 PMC5819730 26424572

[2] Smith RD. Responding to global infectious disease outbreaks: lessons from SARS on the role of risk perception, communication and management. Soc Sci Med. 2006;63(12):3113–3123. 10.1016/j.socscimed.2006.08.004 16978751 PMC7130909

[3] Goh KJ, Tan CT, Chew NK, Tan PSK, Kamarulzaman A, Sarji SA, et al Clinical features of Nipah virus encephalitis among pig farmers in Malaysia. N Engl J of Med. 2000;342(17):1229–1235. 10781618 10.1056/NEJM200004273421701

[4] Looi LM, Chua KB. Lessons from the Nipah virus outbreak in Malaysia. Mal J of Path. 2007;29(2):63–67. 19108397

[5] WHO Ebola Response Team. Ebola virus disease in West Africa—the first 9 months of the epidemic and forward projections. N Engl J Med. 2014;371(16):1481–1495. 10.1056/NEJMoa1411100 25244186 PMC4235004

[6] Upadhyay DK, Sittig DF, Singh H. Ebola virus disease US Patient Zero: lessons on misdiagnosis and effective use of electronic health records. Diagnosis. 2014:1(4):283–287. 26705511 10.1515/dx-2014-0064 PMC4687403

[7] Oner AF, Bay A, Arslan S, Akdeniz H, Sahin HA, Cesur Y. Avian influenza A (H5N1) infection in eastern Turkey in 2006. N Engl J Med. 2006;355(21):2179–85. 10.1056/NEJMoa060601 17124015

[8] Filmer D. Fever and its treatment among the more and less poor in sub-Saharan Africa. Health Policy Plann. 2005;20(6):337–346. 10.1093/heapol/czi043 16155065

[9] Mota REM, Lara AM, Kunkwenzu ED, Lalloo DG. Health seeking behavior after fever onset in a malaria-endemic area of Malawi. Am J Trop Med Hyg. 2009;81(6):935–943. 10.4269/ajtmh.2009.08-0361 19996420

[10] Nonvignon J, Aikins MK, Chinbuah MA, Abbey M, Gyapong M, Garshong BN, et al Treatment choices for fevers in children under-five years in a rural Ghanaian district. Malar J. 2010;9:188 10.1186/1475-2875-9-188 20584280 PMC2914057

[11] Novignon J, Nonvignon J. Socioeconomic status and the prevalence of fever in children under age five: evidence from four sub-Saharan African countries. BMC Res Notes. 2012; 5(1):380. 22840190 10.1186/1756-0500-5-380 PMC3502087

[12] Ahorlu CK, Dunyo SK, Afari EA, Koram KA, Nkrumah FK. Malaria‐related beliefs and behaviour in Southern Ghana: Implications for treatment, prevention and control. Trop Med Int Health. 1997;2(5):488–499. 9217705

[13] Nsungwa‐Sabiiti J, Källander K, Nsabagasani X, Namusisi K, Pariyo G, Johansson A, et al Local fever illness classifications: implications for home management of malaria strategies. Trop Med and Int Health. 2004;9(11):1191–1199. 15548315 10.1111/j.1365-3156.2004.01319.x

[14] Hayman DT, Suu-Ire R, Breed AC, McEachern JA, Wang L, Wood JL, et al Evidence of henipavirus infection in West African fruit bats. PLoS One. 2008;3(7):e2739 10.1371/journal.pone.0002739 18648649 PMC2453319

[15] Hayman DT, Wang LF, Barr J, Baker KS, Suu-Ire R, Broder CC, et al Antibodies to henipavirus or henipa-like viruses in domestic pigs in Ghana, West Africa. PloS One. 2011 9 22;6(9):e25256 10.1371/journal.pone.0025256 21966471 PMC3178620

[16] Han HJ, Wen HL, Zhou CM, Chen FF, Luo LM, Liu JW, et al Bats as reservoirs of severe emerging infectious diseases. Virus Res. 2015 7 2;205:1–6. 10.1016/j.virusres.2015.05.006 25997928 PMC7132474

[17] Calisher CH, Childs JE, Field HE, Holmes KV, Schountz T. Bats: important reservoir hosts of emerging viruses. Clin Microbiol Rev. 2006;19(3):531–545. 10.1128/CMR.00017-06 16847084 PMC1539106

[18] Ayivor JS, Gordon C and Ntiamoa-Baidu Y. Protected area management and livelihood conflicts in Ghana: A case study of Digya National Park. Parks; 2013:19(1):37–50.

[19] Braun V, Clarke V. Using thematic analysis in psychology. Qual Res Psychol. 2006; 3:77–101.

[20] Ghana Statistical Service. 2010 Population and Housing Census. 2014. Ghana Statistical Service.

[21] Sarpong N, Owusu-Dabo E, Kreuels B, Fobil JN, Segbaya S, Amoyaw F, et al Prevalence of malaria parasitaemia in school children from two districts of Ghana earmarked for indoor residual spraying: a cross-sectional study. Malar J. 2015;14(1):260. 26109461 10.1186/s12936-015-0772-6 PMC4479317

[22] Chima RI, Goodman CA, Mills A. The economic impact of malaria in Africa: a critical review of the evidence. Health Policy. 2003;63(1):17–36. 12468115 10.1016/s0168-8510(02)00036-2

[23] Onwujekwe O, Uzochukwu B, Dike N, Okoli C, Eze S, Chukwuogo O. Are there geographic and socio-economic differences in incidence, burden and prevention of malaria? A study in southeast Nigeria. Int J. Equity Health. 2009;8(1):45. 20030827 10.1186/1475-9276-8-45 PMC2806339

[24] Wilson ML, Krogstad DJ, Arinaitwe E, Arevalo-Herrera M, Chery L, Ferreira MU, et al Urban malaria: understanding its epidemiology, ecology, and transmission across seven diverse ICEMR network sites. Am J Trop Med Hyg. 2015;93(3):110–123. 26259941 10.4269/ajtmh.14-0834 PMC4574269

[25] Hay SI, Guerra CA, Tatem AJ, Atkinson PM, Snow RW. Opinion—Urbanization, malaria transmission and disease burden in Africa. Nature Rev Microbiol. 2005;3(1):81–90. 15608702 10.1038/nrmicro1069 PMC3130901

[26] Pond BS. Malaria indicator surveys demonstrate a markedly lower prevalence of malaria in large cities of sub-Saharan Africa. Malar J. 2013;12(1):313. 24021162 10.1186/1475-2875-12-313 PMC3848558

[27] Robert V, Macintyre K, Keating J, Trape JF, Duchemin JB, Warren M, et al Malaria transmission in urban sub-Saharan Africa. The Am J Trop Med Hyg. 2003;68(2):169–176. 12641407

[28] Ghana Statistical Service. Ghana Demographic and Health Survey, 2014 Report. Ghana Statistical Service. 2015.

[29] D’acremont V, Kilowoko M, Kyungu E, Philipina S, Sangu W, Kahama-Maro J, et al Beyond malaria—causes of fever in outpatient Tanzanian children. N Engl J Med. 2014;370(9):809–817. 10.1056/NEJMoa1214482 24571753

[30] Ohemeng F, Lawson ET, Ayivor JS, Leach M, Waldman L, Ntiamoa-Baidu Y. Socio-cultural Determinants of Human–Bat Interactions in Rural Ghana. Anthrozoös. 2017: 30(2): 181–194.

[31] Kamins AO, Restif O, Ntiamoa-Baidu Y, Suu-Ire R, Hayman DT, Cunningham AA, et al Uncovering the fruit bat bushmeat commodity chain and the true extent of fruit bat hunting in Ghana, West Africa. Biol Cons. 2011;144(12): 3000–3008. 10.1016/j.biocon.2011.09.003 PMC3323830 22514356

[32] Gilbert AT, Petersen BW, Recuenco S, Niezgoda M, Gómez J, Laguna-Torres VA, et al Evidence of rabies virus exposure among humans in the Peruvian Amazon. The Am J of Trop Med and Hyg 2012:87(2):206–15. 22855749 10.4269/ajtmh.2012.11-0689 PMC3414554

[33] Hughes JM, Wilson ME, Luby SP, Gurley ES, Hossain MJ. Transmission of human infection with Nipah virus. Clin Infect Dis. 2009;49(11):1743–48. 10.1086/647951 19886791 PMC2784122

[34] Chin RF, Neville BG, Scott RC. Meningitis is a common cause of convulsive status epilepticus with fever. Arch Dis Child. 2005;90(1):66–69. 10.1136/adc.2003.038844 15613516 PMC1720095

