# Supplementary material for: Local classifications of fever and treatment sought among populations at risk of zoonotic diseases in Ghana
Source: PLoS One. 2018 Aug 23;13(8):e0201526. doi: 10.1371/journal.pone.0201526 (PMC6107132; doi:10.1371/journal.pone.0201526)
Supplement: S1 Appendix — (DOCX) [file pone.0201526.s001.docx]

**S1 Appendix. Questionnaire of the Study**

**DYNAMIC DRIVERS OF DISEASES CONSORTIUM**

**Ghana Case Study – Bats and henipaviruses**

*This questionnaire seeks information on human interactions with bats. The questions relate to decisions, use, attitudes and perceptions regarding bats in general. Information gathered will be used strictly for academic purposes, as well as to inform government policies that affect bats and their ecosystems. If at any point you are not comfortable with the questions being asked please inform the interviewer immediately. You are free to end the interview at any point. This study is carried out by staff of the University of Ghana, Legon.*

*Community:* ........................................................... *Interviewer:...............................................*

**SECTION A: SOCIO-DEMOGRAPHIC INFORMATION**

*This section is intended to collect basic data on the socio-demographic characteristics of respondents in the study areas.*

What is your highest level of education? ....................................................................................

1. Gender of respondent 1= Female 2=Male
2. Age of respondent 1= <15 2= 15-25 3=26-35 4=36-45 5= >45
3. Do you live alone? 1=Yes 2=No
4. If you do not, how many people live here (household size)?.............................
5. What is your marital status? 1=Yes 2=No
6. Do you have children? 1=Yes 2=No
7. Where do you come from (hometown)? ..............................................................
8. If yes to Q6, how many children do you have? .................Please state their sex, ages and highest education

| Children | Sex | Age | Highest Education |
| --- | --- | --- | --- |
| 1 |  |  |  |
| 2 |  |  |  |
| 3 |  |  |  |
| 4 |  |  |  |
| 5 |  |  |  |
| >6 |  |  |  |

1. How long have you lived/worked in this area? …………………………………………………...............
2. What illness has the family experienced recently?

........................................................................................................................................................................................................................................................................................................................................................................................................................................................................................................................

1. Has anyone died recently in your family? If so, of what illness?

................................................................................................................................................................................................................................................................................................................................................

1. Do you (or others in the household) ever experience fevers? How often and what do you do about it?

.......................................................................................................................................................................................................................................................................................................................................................................................................................................................................................................................

**SECTION B**

**ASSESSMENT OF GENERAL LIVING CONDITIONS**

*The aim of this section is to collect detailed information on the general living conditions of respondents- housing, employment and income and how these influence bat-human interactions.*

1. . Who owns the house you live in?

1= Self 2= Children 3= Other family members (Please specify) .................................................. 4=Rented 5= Other (please specify) ……………………..

1. What is the main material used in building the walls of your house?

1=Mud/Mud bricks 2=Burnt bricks 3=Wood/Bamboo 4=Iron Sheets 5=Cement/Concrete 6=Other (please specify): .........................................................

1. What is the main material used to roof your house?

1=Mud 2=Thatch 3=Wood 4=Iron Sheets 5=Cement/Concrete 6=Roofing Tiles 7=Other (please specify): ............................................

1. I would like to ask you a few more questions about your home and your assets. Do you have… ?

|  | Yes | No | If yes, number |
| --- | --- | --- | --- |
| Bicycle |  |  |  |
| Motorbike |  |  |  |
| Car |  |  |  |
| Refrigerator |  |  |  |
| Mobile phone |  |  |  |
| Internet access |  |  |  |
| Television |  |  |  |
| Radio |  |  |  |
| Sewing machine |  |  |  |
| Plots of land |  |  |  |

Please list all the various activities that earn you and your household money?

.......................................................................................................................................................................................................................................................................................................................................................................................................................................................................................................................

Do you undertake these activities throughout the year? Please explain.

................................................................................................................................................................................................................................................................................................................................................

Which of these activities bring you close to the bats in this area? How?

................................................................................................................................................................................................................................................................................................................................................

Could you rank the activities you have listed according to season and in order of income importance?

........................................................................................................................................................................................................................................................................................................................................................................................................................................................................................................................

1. Do you have any external sources of income? 1= Yes 2= No

If yes, from where?................................................................................................................

1. How many of the following animals (livestock/poultry/pets) does the household own?

| Livestock | Current  number | Where they are kept | Where they roam during the day | What they feed on | Who takes care of them |
| --- | --- | --- | --- | --- | --- |
| Goats |  |  |  |  |  |
| Sheep |  |  |  |  |  |
| Cattle |  |  |  |  |  |
| Pigs |  |  |  |  |  |
| Turkeys |  |  |  |  |  |
| Chickens |  |  |  |  |  |
| Guinea fowls |  |  |  |  |  |
| Ducks |  |  |  |  |  |
| Grass cutters |  |  |  |  |  |
| Rabbits |  |  |  |  |  |
| Cats |  |  |  |  |  |
| Dogs |  |  |  |  |  |
| Others |  |  |  |  |  |

1. Do you have access to toilet facilities?

1=Yes 2= No

If *yes*, which type?

1=KVIP 2=Pit latrine 3= Water closet 4=Other ............................

1. What is your major source of drinking water?

1= Pipe 2=Borehole 3=Well 4=Stream 5= Harvested rain water

6=Other ...................................

Have you ever seen bats around these water sources or drinking from them?

........................................................................................................................................................................................................................................................................................................................................................................................................................................................................................................................

**SECTION C**

*The aim of this section is to examine the various livelihood activities and how these that bring people in contact with bats. It also assesses levels of interaction with bats and how this affects their attitudes towards bats.*

1. Are you a farmer? 1=Yes 2=No (If no please move to question

If yes, how many farms do you farm on? ................................................. ..........

1. Would you classify them as commercial or peasant farms? .....................................
2. What is your status on the land?

1=Labourer 2=Family land 3= Rent 4= Owner 5= Other.............................

1. How many types of crops do you grow?

| **Type of crop** | **P** | **S** | **M_c** | **Type of crop** | **P** | **S** | **M_c** | **Cowpea** | **P** | **S** | **M_c** |
| --- | --- | --- | --- | --- | --- | --- | --- | --- | --- | --- | --- |
| Avocado Pear |  |  |  | Onion |  |  |  | Yam |  |  |  |
| Bananas |  |  |  | Oranges |  |  |  | Groundnut |  |  |  |
| Beans |  |  |  | Pawpaw |  |  |  | Leafy vegetables (specify) |  |  |  |
| Cassava |  |  |  | Pepper |  |  |  | Maize |  |  |  |
| Cocoa |  |  |  | Pineapple |  |  |  | Mango |  |  |  |
| Cola nut |  |  |  | Plantain |  |  |  | Oil Palm |  |  |  |
| Cocoyam |  |  |  | Rice |  |  |  | Cashew |  |  |  |
| Okro |  |  |  | Rubber |  |  |  | Other fruits |  |  |  |
| Sugar Cane |  |  |  | Timber |  |  |  | Other grain |  |  |  |
| Sweet Potatoes |  |  |  | Garden egg |  |  |  | Other vegetables |  |  |  |

P= perennial S= Seasonal M*_c= Months cultivated*

1. Why do you grow those crop combinations?

....................................................................................................................................................................................................................................................................................................................

1. Can you estimate the size of the farm? (Note if it is local acre or standard acre) .......................................................................................................................................................................
2. Do you see bats on your farm(s)? ..................................................................................................................
3. Do the bats feed your farm products? If so which part of the plant do they feed on?

.......................................................................................................................................................................

What do you do with fruit which has been damaged by bats ...............................................................................................................................................................................................................................................................................................................................................

1. Does it affect your output? ...........................................................................................................................
2. What would you do to the bats if you saw them feeding on your farm? .......................................................................................................................................................................
3. Are you a hunter? 1= Yes 2=No
4. If yes, how often do you go hunting? Days/weeks/months......................................................................................................................................
5. Which animals do you often catch and why?

................................................................................................................................................................................................................................................................................................................................................

1. Do you hunt bats 1=Yes 2= No (If No probe for reasons)...........................................................................
2. If *yes*, how often do you hunt bats? Days/weeks/months ............................................................................
3. How many bats do you catch each time you hunt? .......................................................................................
4. Where do you hunt your bats? ......................................................................................................................................................................
5. What method or methods do you use to catch/kill a bat?

.......................................................................................................................................................................................................................................................................................................................................................................................................................................................................................................................

1. Do you sell them or they are just for consumption?......................................................................................
2. If you sell them how much did you sell your bats the last time? [GHC] .....................................................
3. How far do you have to travel to sell the bats and how do you get them there? .......................................................................................................................................................................
4. To whom do you sell your bats? (Occupation and location).

.......................................................................................................................................................................

........................................................................................................................................................................

1. Do your children bring some bats? If so how often?

...............................................................................................................................................................................................................................................................................................................................................

1. Do you sometimes buy/sell bat meat? Why do you so answer?

.......................................................................................................................................................................................................................................................................................................................................................................................................................................................................................................................

1. In what form do you obtain the bat meat?

................................................................................................................................................................................................................................................................................................................................................

1. If you sell bats how many customers ask for bat meat each day? .......................................................................................................................................................................
2. How much do you sell a bat for? [GHC]......................................................................................................
3. Where do the bats come from (geographically)?...........................................................................................
4. How does the time of year affect your income from bats? ...........................................................................
5. Do you know of traditional methods of curing diseases? Please give examples of diseases.

........................................................................................................................................................................

........................................................................................................................................................................

1. Do you eat bats? 1= Yes 2=No

*If yes:*.............................................................................................................................................................

1. How often do you eat bat meat? 1. Daily 2. 2-5 times a week 3. Weekly 4.Monthly 5.Less often
2. Why do you eat bat meat? (probe for reasons e.g. availability, cost, taste, health benefits etc) ...............................................................................................................................................................................................................................................................................................................................................
3. In what state do you commonly get bat meat?

1=Fresh 2=Already butchered 3=Smoked 4= Other (please specify)...........................................................

1. How does the time of year affect your consumption of bats? .......................................................................................................................................................................
2. Do you sometimes prepare the bat yourself? And how do you do it?

.................................................................................................................................................................

.................................................................................................................................................................

1. In your view which of these bat-related activities can be risky?

| Activity | No threat | Small Threat | Significant threat | Serious threat | Reasons |
| --- | --- | --- | --- | --- | --- |
| Butchering/preparing |  |  |  |  |  |
| Eating |  |  |  |  |  |
| Hunting |  |  |  |  |  |
| Cooking |  |  |  |  |  |

1. Do you think that people’s taste for bats have increased or decreased over the last 5 years?

..................................................................................................................................................................

...................................................................................................................................................................

1. Do you encounter fewer or more bats around now than you did five years ago?

...................................................................................................................................................................

...................................................................................................................................................................

1. Can you list 3 benefits of bats? 1= Yes 2=No
2. Can you list 3 things you do not like about bats? 1= Yes 2=No
