# Supplementary material for: Local classifications of fever and treatment sought among populations at risk of zoonotic diseases in Ghana
Source: PLoS One. 2018 Aug 23;13(8):e0201526. doi: 10.1371/journal.pone.0201526 (PMC6107132; doi:10.1371/journal.pone.0201526)
Supplement: S1 File — (DOC) [file pone.0201526.s002.doc]

S2. File. STROBE Statement—checklist of items that should be included in reports of observational studies

|  | Item No | Recommendation |
| --- | --- | --- |
| **Title and abstract** | 1 | Title**: Local classifications of fever and treatment sought among populations at risk of zoonotic diseases in Ghana**  Abstract: In the past four decades, there has been an increase in the occurrence of zoonotic diseases. Some of the outbreaks have been devastating because of the inability of individuals and health workers to identify the diseases early. Generally, most zoonotic diseases are heralded by a fever. While fevers are common, they are often the symptoms of different diseases. This paper explores how a population at risk of bat-borne zoonotic diseases identify fevers, and what treatments they seek when they get fevers. The data are from focus group discussions and a survey of three communities in the Brong Ahafo, Volta and Greater Accra regions in Ghana. The quantitative data were analysed using descriptive statistics while the qualitative data was analysed using thematic analysis. The findings indicate that the perceived causes of fever differ from the biomedical view. While orthodox treatment was the preferred choice for most participants, rural dwellers utilised traditional medicine more than their urban counterparts did. Though there is no record of bat-borne zoonotic disease in Ghana, the findings could be used as a proxy to indicate how populations at risk would respond in the event of a spillover of a disease. We recommend that educational campaigns on zoonotic diseases should target rural dwellers, mainly farmers, who tend to be most at risk of bat-borne diseases. |
|  |  |  |
| Introduction | | |
| Background/rationale | 2 | Zoonotic diseases are on the rise globally. Bats have been found to be carriers of many disease pathogens. Generally, fever is a common symptom of many diseases and common in Ghana. The challenge with many disease outbreaks is the ability to correctly diagnose for prompt treatment and prevention. The rationale for this study is to explore how people distinguish between different kinds of fevers and what treatment they seek when they are sick. The findings from this study would be useful in the design of educational programmes on potential disease outbreaks. |
| Objectives | 3 | The objectives of the study are to explore how people who live close to bat roost classify fevers and the treatment they seek when they experience fevers. |
| Methods | | |
| Study design | 4 | The study used survey and focus group discussions to collect data. Participants were recruited through a simple random sampling. |
| Setting | 5 | The study areas were made of three communities that live in close proximity to large bat roost from three regions in Ghana. The data were collected between January and August 2013. |
| Participants | 6 |  |
|  |  |  |
| Variables | 7 | This is not applicable. |
| Data sources/ measurement | 8* | Not applicable*.* |
| Bias | 9 | There were no bias in the study. |
| Study size | 10 | There were three communities included in the study we decided on a hundred participants from each community. This is because two of the communities are rural areas with a population of not more than 5,000 each. The focus group discussion were based on the sexes—male and female. |
| Quantitative variables | 11 | The data were coded and uploaded unto the SPSS version 21. |
| Statistical methods | 12 | (*a*) The Statistical software, SPSS version 21 was used to analyse the data. Simple frequencies to describe the variables were generated from the SPSS data. Chi-square were also used to find the relationships between variables. |
|  |  | (*b*) *b* to *d* is not applicable. |
|  |  |  |
|  |  |  |
|  |  |  |

| Results | | |
| --- | --- | --- |
| Participants | 13* | (a) 340 people took part in the survey and 136 people took part in the focus group discussions. |
|  |  |  |
|  |  |  |
| Descriptive data | 14* | (a) Give characteristics of study participants (eg demographic, clinical, social) Sex: female— 164; males— 174. Age: 14-50+. Education: No education—15.9%; Primary—10%; JHS/SHS—34.4%; Tertiary—21.5%. |
|  |  |  |
|  |  |  |
| Outcome data | 15* | *Cohort study*— Not applicable. |
|  |  | *Case-control study—Not applicable.* |
|  |  | *Cross-sectional study—Not applicable* |
| Main results | 16 | (*a*) The participants reported they experience many diseases such as malaria, fever, headaches, rheumatism and skin diseases. More than 70% said they have experienced fever. The participants also identified two types of fevers, normal fever and high fever. The results suggested a statistically significant relationship between economic activity (farmers and non-farming activities) and incidence of fever (X^2^= 4.20, N= 284, df= 1, p = 0.039). The findings also indicate that majority of the participants sought hospital for treatment. However, rural dwellers are more likely to use herbal medicine more than city dwellers. |
|  |  |  |
|  |  |  |
| Other analyses | 17 |  |
| Discussion | | |
| Key results | 18 | The findings showed that fever was caused by eating un-ripen mangoes, staying for too long in the sun, and eating fresh yams. Two types of fevers were identified by the participants. These were normal fever and high fever. We also found out that farmers tend to experience fevers more than other category of workers. The treatment sought by the participants were going to the hospital, buying drugs off the counter and using herbal medicine. While majority of the participants attended hospital when sick, rural dwellers tend to use herbal medicine more than the urban dwellers. |
| Limitations | 19 | The limitations of this study is that we were not able to determine the actual incomes of participants. This hindered our ability to analyse the relationship between income and the experience of fever. |
| Interpretation | 20 | The results indicate that how people perceive the causes of some diseases is different from the biomedical interpretation of the disease. |
| Generalisability | 21 | The findings cannot be generalised to the whole Ghanaian population because the study was limited to three communities that live close to large bat roost. |
| Other information | | |
| Funding | 22 | This work was supported by the Ecosystem Services for Poverty Alleviation (ESPA). NERC project no. NE-J00 1570-1, 192. The funders have no role in the conceptualisation, execution and publication of the study. |

*Give information separately for cases and controls in case-control studies and, if applicable, for exposed and unexposed groups in cohort and cross-sectional studies.
